# Supplementary material for: The effectiveness of community engagement using M-Mama champions in improving awareness of obstetric danger signs, birth preparedness and complication readiness among pregnant women in Bahi, Dodoma: A cluster randomized pragmatic implementation trial
Source: PLOS Glob Public Health. 2025 Apr 8;5(4):e0004315. doi: 10.1371/journal.pgph.0004315 (PMC11978080; doi:10.1371/journal.pgph.0004315)
Supplement: Attachment — Submitted filename: Response to Reviewers.docx 25.pdf [file pgph.0004315.s004.pdf]

ALEX SANGA,
SCHOOL OF NURSING AND PUBLIC HEALTH,
UNIVERSITY OF DODOMA,
P. O. BOX 259, DODOMA.

1st FEBRAURY, 2025.

Dear Editor,

REF: <u>MANUSCRIPT RESPONSE TO REVIEWERS</u>

We thank the reviewers for their invaluable time and energy spent reviewing our manuscript and providing significant comments, which we have addressed accordingly.

Moreover, in this letter, we have provided a point-by-point response in italics to each comment as it has been addressed in the manuscript. To this point, we believe that the manuscript meets PLOS Global Public Health's publication criteria rendering it suitable for publication.

A.sanga

Dr. Alex Sanga (MD, MSc.)

On behalf of all authors

Thanks for requesting that I review this paper.
The study investigates the effectiveness of community engagement through M-Mama Champions in raising awareness of obstetric danger signs and improving birth preparedness and complication readiness among pregnant women in Dodoma, Tanzania.

The research is relevant and aligns with global maternal and child health priorities, especially in rural areas where maternal health education is critical to reducing maternal and child mortality.

Areas requiring reviews that could strengthen the study

Choice of facility
The study is conducted in rural wards of Bahi, Dodoma, Tanzania. Facility selection includes four wards, each with at least two healthcare facilities (either health centers or dispensaries). The choice of these rural facilities is well justified given the focus on reaching underserved populations where the risk of maternal mortality is higher. The inclusion of both health centers and dispensaries appears to address variability in resources and access within the community, providing a representative look at maternal healthcare challenges in rural settings.

- Clarify Facility Criteria: The rationale for selecting specific wards with particular facility types could be expanded. For instance, including how facility resources, personnel, or services influenced their selection would highlight how these differences might impact intervention outcomes.

We have provided further clarification to justify the health facilities selection criteria. The justification has gone around the three main criteria for their selection, being located in the rural setting, having records of post-natal mothers who benefited from M-MAMA referral and emergency system. Those wards whose facilities had the largest number of post-natal mothers who could be legible to deliver the intervention were selected. The mixture of health centers and dispensaries was necessary for representativeness of common facilities operating in the rural settings and comparability.

- Explanation of Facility Distribution: Since two wards were randomly assigned to the intervention or control arms, an explanation of how the assignment affected facility distribution (if relevant) would provide additional clarity. If geographic proximity to other healthcare services or resource availability differed among wards, this could have implications for intervention efficacy.

We have explained the health facilities' geographical distribution and the distance from the nearest higher referral point which would confound the intervention outcome. There was a slight difference in distance to the referral point among the selected health facilities which might have affected the efficacy of the intervention.

Study Participants

The study includes pregnant women in their first and second trimesters (gestational age up to 28 weeks). Selection was randomized, with an equal number of participants from each facility

- Inclusion and Exclusion Criteria: The inclusion criteria are clearly defined (first and second trimesters), and the study excludes women with serious health conditions. While the criteria help focus on early pregnancy education, expanding the rationale for excluding women in later trimesters or addressing how this might affect birth preparedness insights would be helpful.

We have provided the rationale for excluding the third-trimester pregnant women. For effective birth preparedness and complication readiness, antenatal care contacts at early pregnancy are encouraged by the World Health Organization, third-trimester pregnant women, were excluded to reduce the attrition rate at end-line data collection, especially among those who will have delivered for they are less likely to show up because at this point won't be pregnant any more, making them a different population. This would affect the conclusion of the intervention outcome.

- Remoteness and Community Awareness: Considering the members of these communities, despite all rural settings, the level of remoteness of each site also plays a significant role in health and social awareness. Generally, less remote areas tend to have greater access to information and services, leading to a higher level of health and social awareness compared to more isolated locations.

We have clarified the remoteness of the selected clusters. The ownership of a working radio as a means of media access and walking distance to the health facility determined the remoteness of the clusters. There was no significant difference between arms after analysis of the collected data on the possible confounders.

Review Bias in Outcome Assessment: Losses to follow-up due to adverse pregnancy events could bias the study findings by excluding cases that might reveal gaps in the intervention's effectiveness or identify additional needs that were not met. This could lead to an overly optimistic assessment of the intervention’s success if only healthier or less complex cases remain in the study.

I would recommend including a mechanism to track reasons for loss to follow-up as this could provide critical insights into whether adverse outcomes are contributing to study dropout. If feasible, a follow-up to gather information on lost cases would strengthen the study's conclusions and provide a more comprehensive picture of maternal health needs in the community.

We have included the mechanism used to track reasons for loss to follow-up after end-line data collection. This included both phone calls by the researcher and the physical visits by the community health workers to trace the reason for attrition.

Limited Intervention Duration and Follow-up: The intervention's duration (one month) may not provide sufficient time to instil lasting changes in awareness and practices. A short follow-up period might capture only the immediate effects of the intervention, without insight into how well these practices are retained over time or during delivery. Given the importance of sustained knowledge and readiness, a follow-up at later stages (e.g., late pregnancy or postpartum) could provide stronger evidence of long-term efficacy and any adjustments needed for sustained impact.

We acknowledge the limited duration of only one month for intervention delivery to evaluation that, doesn't guarantee a long-lasting sustained change in awareness and practices. We, therefore have included it as one of the limitations to the sustained awareness and practice in our study. We have also recommended the evaluation after delivery to determine the sustained interventions in the next similar studies.

Confounding Variables: Though this study was in a rural context- confounding variables, such as socioeconomic status, access to transportation, or cultural beliefs about childbirth, which could impact awareness and preparedness levels were not reviewed for or assessed. it's difficult to determine how much of the improvement in awareness and preparedness is due to the intervention versus other factors.

We have explained the possible confounding variables, whose data were collected and analyzed; socio-demographic (age, education level, marital status, parity, number of ANC visits) and socio-economic (type of living house, drinking water source, livestock ownership, radio ownership, mobile phone ownership, means of transport commonly used and distance to the nearest health facility). However, none was determined to significantly of the outcomes influence either negatively or positively the outcome of interest in this study.
